# Obesity Enhances Non-Th2 Airway Inflammation in a Murine Model of Allergic Asthma

**DOI:** 10.3390/ijms25116170

**Published:** 2024-06-04

**Authors:** Marwa M. E. Mohamed, Yassine Amrani

**Affiliations:** Department of Respiratory Sciences, Clinical Sciences, Glenfield Hospital, University of Leicester, Leicester LE3 9QP, UK; mm1110@leicester.ac.uk

**Keywords:** asthma, obesity, house dust mite, airway inflammation

## Abstract

Obese patients with asthma present with aggravated symptoms that are also harder to treat. Here, we used a mouse model of allergic asthma sensitised and challenged to house dust mite (HDM) extracts to determine whether high-fat-diet consumption would exacerbate the key features of allergic airway inflammation. C57BL/6 mice were intranasally sensitised and challenged with HDM extracts over a duration of 3 weeks. The impact of high-fat-diet (HFD) vs. normal diet (ND) chow was studied on HDM-induced lung inflammation and inflammatory cell infiltration as well as cytokine production. HFD-fed mice had greater inflammatory cell infiltration around airways and blood vessels, and an overall more severe degree of inflammation than in the ND-fed mice (semiquantitative blinded evaluation). Quantitative assessment of HDM-associated Th2 responses (numbers of lung CD4^+^ T cells, eosinophils, serum levels of allergen-specific IgE as well as the expression of Th2 cytokines (*Il5* and *Il13*)) did not show significant changes between the HFD and ND groups. Interestingly, the HFD group exhibited a more pronounced neutrophilic infiltration within their lung tissues and an increase in non-Th2 cytokines (*Il17*, *Tnfa*, *Tgf-b*, *Il-1b*). These findings provide additional evidence that obesity triggered by a high-fat-diet regimen may exacerbate asthma by involving non-Th2 and neutrophilic pathways.

## 1. Introduction

Asthma is a complex disease characterised by airway hyper-responsiveness, airway inflammation and structural changes within the airways known as airway remodelling [1]. Asthma remains a global health problem with an estimated 300 million individuals worldwide affected by the condition, a number which could increase by another 100 million by 2025 [2]. The increased prevalence and incidence of asthma can be explained by genetic, environmental and lifestyle risk factors [3]. The main clinical features of allergic asthma result from the action of different cytokines secreted from activated CD4^+^ T-helper 2 (Th2) cells, which leads to IgE production (IL-4) and mast cell activation, mucus secretion (IL-13), bronchial hyper-responsiveness (IL-13) and eosinophilic lung inflammation (IL-5) [4,5]. Although asthma has been traditionally described as a condition driven by a Th2 inflammatory process, accumulating clinical evidence suggests that the pathogenesis is far more complex and encompasses different clinical phenotypes associated with different inflammatory endotypes including T2 high and T2 low [6,7]. 

Several clinical studies have demonstrated that obesity, characterised by an excessive amount of adipose tissue, represents an important risk factor for developing asthma [8,9,10]. Obesity has been described as a major factor involved in at least two different clinical asthma phenotypes, namely early-onset allergic asthma, which is aggravated by obesity, and late-onset asthma, primarily seen in non-atopic female patients, which appears to be induced by obesity [6,7]. In addition, obese asthmatics tend to have a more severe form of the disease as these patients suffer from more frequent exacerbations [11], respond poorly to conventional asthma therapies including corticosteroids [12], and experience more asthma-related hospitalisations [13]. The underlying mechanisms that link obesity and poor asthma outcomes have not been clearly established. Several preclinical studies performed in murine models of allergic asthma have indeed shown the significant impact of obesity on allergic eosinophilic inflammation [14,15,16,17,18,19,20]. Indeed, a previous study performed using extracts from the house dust mite (HDM) Dermatophagoides pteronyssinus, a common aeroallergen associated with asthma and rhinitis [21], showed a completely different impact of obesity on the profile of allergic airway inflammation. In contrast to the studies that have used mice sensitised and challenged to ovalbumin, this study showed that HDM-induced eosinophilic inflammation was not affected by obesity, while lung accumulation of macrophages was increased [22]. Another study that used allergen extracts from Dermatophagoides farina (Der f) observed that obesity increased HDM challenge and was associated with neutrophilic but not eosinophilic inflammation in intranasally sensitised BALB/c mice [23]. This is an interesting observation as obesity in asthmatic patients tends to be associated with more Th1/Th17 and decreased eosinophilia in the airways [11]. Together, these observations show that the translation value of the preclinical models assessing the impact of obesity on asthma may be improved by using clinically relevant allergens (HDM allergens vs. ovalbumin) and routes of sensitisation (inhalation vs. subcutaneous and/or intraperitoneal sensitisation/challenge).

In this study, we examined the impact of obesity, driven by a high-fat-diet (HFD) regime, in a murine model of allergic airway inflammation triggered solely by the intranasal administration of HDM during sensitisation and challenge. We found that obesity following a HFD regime significantly increased allergen-induced pulmonary inflammation, which correlated with the extent of neutrophilic infiltration. Interestingly, while no changes in HFD were seen on HDM-induced changes in markers of Th2 response (eosinophilic and CD4^+^ T cell infiltration, levels of allergen-specific IgE, and the expression of *Il5* and *Il13* cytokines), obesity markedly increased cytokines such as *Tnfa*, *Il-1b*, *Tgf-b* and *Il17* when compared to HDM-exposed and -sensitised lean mice. These studies show that obesity exacerbates allergic airway inflammation by increasing non-Th2 immune pathways. 

## 2. Results

### 2.1. A High-Fat Diet Is Associated with Obesity as Evidenced by the Increased Body Weight

**Figure 1:**
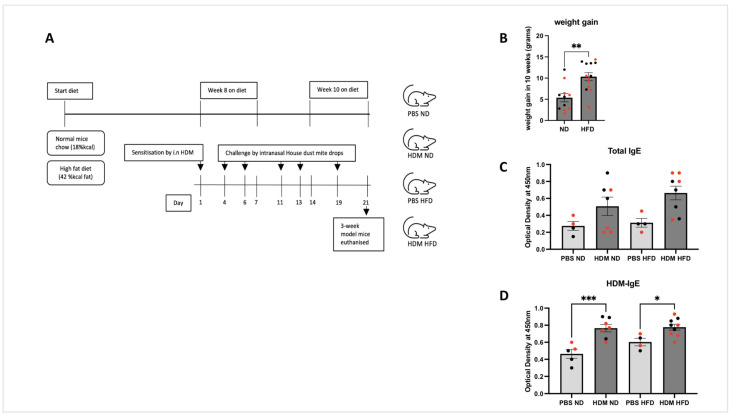
**Experimental protocol for the induction of allergic asthma in obese and lean mice.** (**A**) C57BL/6 mice were fed either a high-fat diet (HFD) (*n* = 13) or normal chow (ND) (*n* = 11) for 10 weeks. On week 8, day 1 mice were sensitised by intranasal house dust mite (HDM) extract (25 µg of *D. Pteronyssinus*) then followed by five doses of intranasal HDM. Control mice received PBS only. PBS ND are mice on a normal diet and received intranasal administration of PBS buffer. HDM ND are mice on a normal diet and received intranasal administration house dust mite. PBS HFD are mice on a high-fat diet and received intranasal administration of PBS buffer. HDM HFD are mice on a high-fat diet and received intranasal administration of HDM. (**B**) Obesity in HFD for 10 weeks was confirmed by the increase in body weight measured at 10 weeks (** *p* = 0.0015). (**C**) Levels of total serum IgE did not significantly change with sensitisation. (**D**) Allergen sensitisation was confirmed by the increased serum levels of HDM-specific IgE (*** *p* < 0.001, * *p* < 0.05). Data represent the mean ± SEM (*n* = 4–9 per group). An unpaired *t*-test was used to compare between groups, with each circle representing a different mouse; females are marked in red.

Male and female C57BL/6 mice that were 14–15 weeks of age were fed either a high-fat diet (HFD) (42% calories from fat, TD.88137, Envigo, Madison, WI, USA) or their normal maintenance diet (ND) (Teklad 18% protein, Envigo) for 10 weeks (Figure 1A). Figure 1B shows that mice fed on HFD had gained significantly more body weight (mean = 10.34 ± 3.39 g) than those in the normal diet (ND) group (mean = 5.38 ± 3.27 g) over the 10-week period (** *p* < 0.01). This suggests that the diet rich in fat induced a profound weight gain in these mice.

### 2.2. Allergic Sensitisation Is Induced by Intranasal Challenge with HDM in Both ND and HFD Mice

Serum samples from allergen-sensitised and -challenged mice were assayed for total and HDM-specific IgE as described in (Post et al., 2012 [24]). Sensitisation/challenge with HDM versus PBS control caused an increase in the titres of total IgE (Figure 1C, difference did not reach significance) and HDM-specific IgE (Figure 1D, *** *p* < 0.001; * *p* < 0.05) although comparable levels were seen between sensitised/challenged ND and HFD mice. This suggests that diet did not impact the sensitisation/challenge process induced by HDM.

### 2.3. Impact of HFD on Allergen-Induced Pulmonary Inflammation and Mucus Hypersecretion

**Figure 2:**
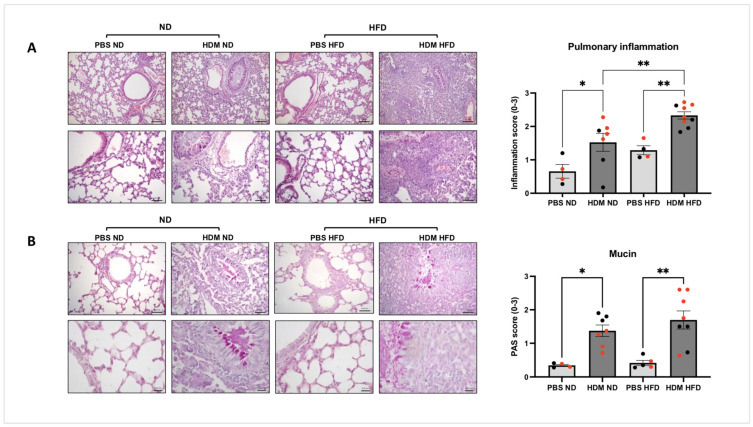
**Pulmonary inflammation and mucus secretion in HDM-sensitised and -challenged obese (HFD) and lean (ND) mice**. C57BL/6 mice fed with either ND or HFD were sensitised and challenged with intranasal HDM before performing a histological evaluation of inflammatory cell infiltration ((**A**), ** *p* < 0.01 * *p* < 0.05) or mucus secretion assessed using Periodic acid-Schiff staining ((**B**), ** *p* < 0.01, * *p* < 0.05) in the lung sections using a score based on a subjective scale from 0 to 3. Data represent the mean ± SEM (*n* = 4–9 per group). ANOVA was used to compare between groups, with each circle representing a different mouse; females are marked in red. (**A**), bar = 0.1 and 0.05 mm. (**B**), bar = 0.05 and 0.02 mm.

Allergen-induced pulmonary inflammation was assessed by measuring the degree of inflammatory cell infiltrates in three main locations—parenchymal, peribronchiolar and perivascular areas—using a subjective score based on a scale from 0 to 3 as reported in [25,26,27]. As expected, compared to the PBS control groups, HDM challenge led to a significant increase in pulmonary inflammation in both ND mice (mean score = 1.525, * *p* < 0.05) and HFD mice (mean score = 2.330, ** *p* < 0.01). It is important to note that the degree of HDM-induced pulmonary inflammation was more pronounced in the HFD group compared to that seen in the ND group (** *p* < 0.01, Figure 2A). Although allergen-challenged female mice appeared to have more pulmonary inflammation compared to male mice in the ND group, this difference did not reach statistical significance (Figure 2A, *p* = 0.084). We next investigated the impact of HDM on goblet cell hyperplasia and the consecutive increase in mucus production in both diet groups using the PAS stain. Figure 2B shows that compared to the PBS groups, HDM challenge led to a significant increase in mucus hypersecretion in the bronchi of both ND mice (mean score = 1.375, * *p* < 0.05) and HFD mice (mean score = 1.847, ** *p* < 0.01). The degree of HDM-induced mucus hypersecretion was not different between the HFD and ND groups (*p* = 0.257, Figure 2B). When looking at the PAS staining data, it was interesting to note that allergen-induced mucus secretion was more pronounced in sensitised and challenged female mice fed with HFD when compared to responses seen in female mice fed with ND (Figure 2B, ** *p* < 0.01). In agreement with other groups [28], we did not see that gender difference was affecting the allergic airway responses in non-obese conditions that was previously reported by others who showed that allergen-induced airway remodelling, and mucus secretion was more pronounced in sensitised and challenged female compared to male mice [29,30]. Nonetheless, our finding is in line with some clinical evidence showing that the association between obesity and asthma may be greater in women compared to men [31]. Additional studies are clearly needed to further confirm this hypothesis and investigate the mechanisms linking airway remodelling and gender in asthma. Together, these data show that HFD is associated with greater allergic inflammation in the lungs. 

### 2.4. Impact of HFD on Allergen-Induced Pulmonary Infiltration of Inflammatory Cells

**Figure 3:**
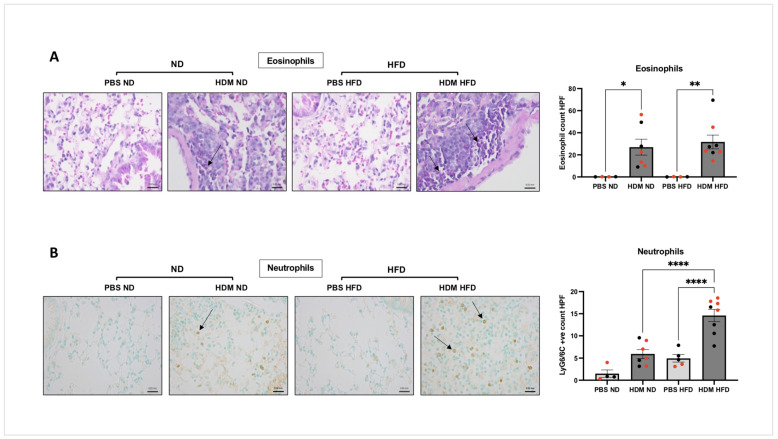
**Granulocytic cell infiltration in HDM-sensitised and -challenged obese (HFD) and lean (ND) mice**. C57BL/6 mice fed with either ND or HFD were sensitised and challenged with intranasal HDM before assessing the abundance of eosinophils ((**A**), ** *p* < 0.01 * *p* < 0.05) or neutrophils ((**B**), **** *p* < 0.0001) in the lung sections using actual count per HPF (high power field, 400×). Data represent the mean ± SEM (*n* = 4–8 per group). ANOVA was used to compare between groups, with each circle representing a different mouse; females are marked in red. Bar = 0.02 mm.

We next looked at the impact of diet on allergen-induced pulmonary infiltration of neutrophils and eosinophils in lung sections. Histological assessment of the lungs shows that compared to the PBS groups, HDM challenge led to a significant increase in eosinophil number in the bronchi of both ND mice (26.96 ± 19.09, * *p* < 0.05) and HFD mice (31.68 ± 17.59, ** *p* < 0.01, Figure 3A) with no significant different between the ND and HFD groups (*p* = 0.911). Interestingly, compared to the PBS group, neutrophilic pulmonary infiltration following allergen challenge was significantly increased in HFD mice (14.59 ± 3.9, **** *p* < 0.0001, Figure 3B) but not in ND mice (*p* = 0.075). In addition, allergen-sensitised and -challenged mice fed HFD had a statistically greater number of lung-infiltrated neutrophils comparing to ND mice (**** *p* < 0.0001, Figure 3B). 

**Figure 4:**
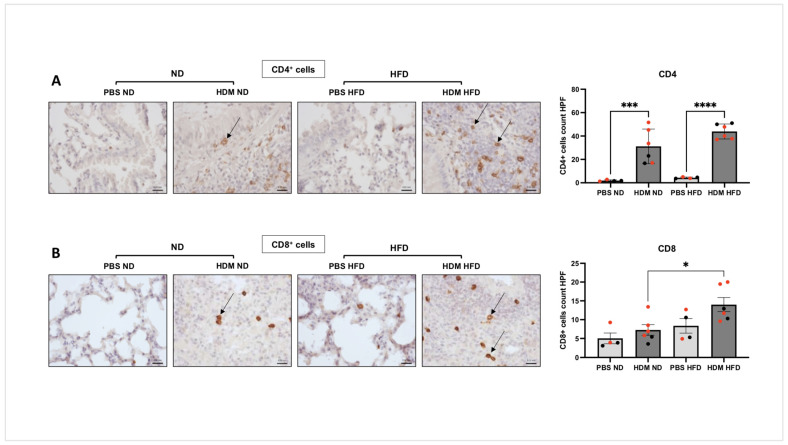
**Lymphocytic cell infiltration in HDM-sensitised and -challenged obese (HFD) and lean (ND) mice**. C57BL/6 mice fed with either ND or HFD were sensitised and challenged with intranasal HDM before assessing the abundance of CD4^+^ cells ((**A**), **** *p* < 0.0001, *** *p* = 0.001) or CD8^+^ cells ((**B**), * *p* < 0.05) in the lung sections using actual count per HPF (high power field, 400×). Data represent the mean ± SEM (*n* = 4–6 per group). ANOVA was used to compare between groups, with each circle representing a different mouse; females are marked in red. Bar = 0.02 mm.

**Figure 5:**
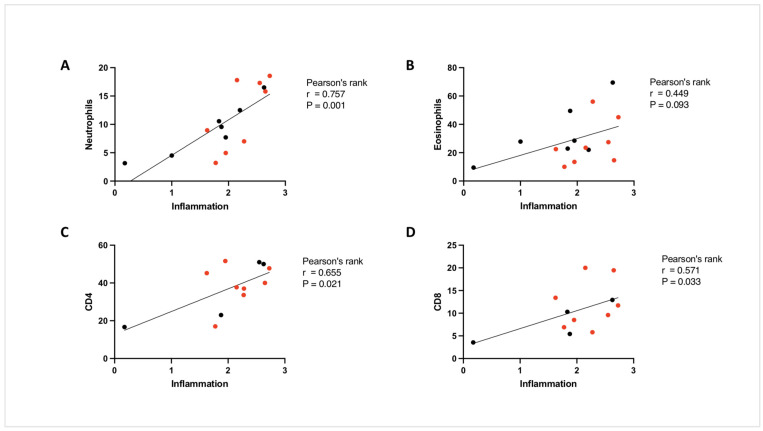
Correlation between the number of granulocytes and lymphocytes with airway inflammation. Pearson’s rank correlation coefficient was determined to assess the relationship between the number of neutrophils (**A**), eosinophils (**B**), CD4^+^ T cells (**C**) and CD8^+^ T cells (**D**) and the inflammation score in the lungs of HDM-sensitised and -challenged mice on normal diet (ND) and high-fat diet (HFD); females are marked in red.

Figure 4A shows that while lung infiltration of CD4^+^ T cells was also significantly increased by HDM in both the ND (31.18 ± 14.81, *** *p* < 0.001) and HFD groups (43.92 ± 6.38, **** *p* < 0.0001), the number of infiltrated CD8^+^ T cells were only increased in the HFD group (14.0 ± 4.59, * *p* < 0.05, Figure 4B). In addition, we found a strong association between the number of pulmonary neutrophils and the extent of pulmonary inflammation using Pearson’s correlation (r = 0.757, *** *p* = 0.001, Figure 5A). Interestingly, the correlation between eosinophils and pulmonary inflammation did not reach significance (r = 0.449, *p* = 0.09, Figure 5B). The numbers of CD4^+^ and CD8^+^ T cells showed a significant positive correlation with pulmonary inflammation (r = 0.65 and 0.57, respectively, ** *p* < 0.01, Figure 5C,D). Together, these data show that HFD is associated with a greater pulmonary inflammation in the lungs of HDM-sensitised and -challenged mice that likely result from T cell-driven neutrophilic infiltration.

### 2.5. The Impact of HFD on Inflammatory Mediators

**Figure 6:**
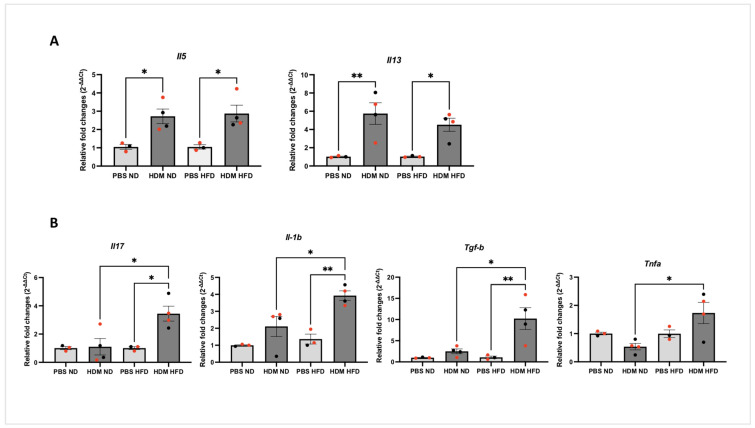
**The expression of inflammatory mediators in HDM-sensitised and -challenged obese (HFD) and lean (ND) mice**. C57BL/6 mice fed with either ND or HFD were sensitised and challenged with intranasal HDM before measuring the relative expression of Th2 cytokine ((**A**), ** *p* < 0.01 * *p* < 0.05) or non-Th2 inflammatory mediators ((**B**), ** *p* < 0.01 * *p* < 0.05) in the lung homogenate using qPCR. Data represent the mean ± SEM (*n* = 3–4 per group). ANOVA was used to compare between groups, with each circle representing a different mouse; females are marked in red.

We next looked at the impact of HDM on the expression of inflammatory gene expression in lung homogenates using qPCR assays. We found that HDM significantly increased the expression of various Th2 cytokines *Il5* (* *p* < 0.05) and *Il13* (* *p* < 0.05, ** *p* < 0.01), although no significant difference was observed between HFD and ND mice (Figure 6A). Among the cytokines that were only upregulated by HDM in the HFD lungs compared to both the PBS and ND groups were *Il17*, *Tgf-b*, *Il-1b*, and *Tnfa* (* *p* < 0.05, Figure 6B). Cytokine gene expression was evaluated in total lung homogenates using a TRIzol-based technique as described by [32,33,34,35].

## 3. Discussion

In this report, we assessed the impact of diet-induced obesity on allergic airway inflammation in a mouse model (C57BL/6 strain) sensitised and challenged with house dust mite (HDM), a common aeroallergen in 50–85% of asthmatic patients [36]. We found that the degree of HDM-induced pulmonary inflammation was significantly greater in obese mice compared to lean mice. HDM-induced pulmonary inflammation correlated with the extent of neutrophilic infiltration. Interestingly, HDM-induced Th2 responses (eosinophilic and CD4^+^ T cell infiltration, levels of allergen-specific IgE, and Th2 cytokines gene expression *Il5* and *Il13*) were not different between lean and obese mice. In contrast, cytokines such as *Tnfa*, *Il-1b*, *Tgf-b* and Th17 response (*Il17*) were significantly increased in obese mice compared to lean mice. These data provide further evidence of a role of abnormal Th17–neutrophil axis in obese allergic asthma. This hypothesis was recently confirmed in an elegant study performed in a large, well-characterised cohort of German patients showing that obesity in severe asthma was associated with elevated neutrophil numbers while blood eosinophils were independent of obesity [37].

The impact of obesity on allergic asthma in preclinical studies has been previously examined by several groups although their diet-induced obesity protocols varied a lot with regard to both % calories from fat (ranging from 40 to 60% calories from fat) and the treatment duration varying from 8 to 30 weeks with a significant difference on body mass gain [14,15,16,17,18,22,38]. Our approach of diet-induced obesity consisting of mice fed with a HFD with 42% calories from fat for 10 weeks significantly increased weight gain by 2-fold when compared to the ND group (Figure 1A). Our observation is somewhat consistent with the general conclusion that feeding mice with HFD (with at least 40% of calories from fat) and for periods > 10 weeks results in obesity independently of the sex and strain of the mice (i.e., C57BL/6 and BALB/c). Using this model, we found that HDM-induced lung inflammation was more severe in obese mice, a finding consistent with previous studies using either a OVA [15,39,40,41] or HDM model of allergic asthma [22,23]. 

It was interesting to note that the HFD regime on its own was associated with a trend toward increasing inflammatory scores and neutrophil counts in the lungs, even though this did not reach statistical significance. The relevance of such observation is not clear but a similar non-significant increase in neutrophils in the lungs of HFD-fed mice vs. ND-fed mice was also reported by Fricke and colleagues using a sample size of *n* = 5 animals per group [42]. The authors suggested that increased levels of IL-1β by HFD were likely one of the mechanisms driving lung dysfunction in their obese mice. Indeed, IL-1β was reported to promote lung neutrophilia in a mouse model of respiratory virus-induced asthma exacerbation [43].

Our current study shows that HFD-induced obesity exacerbates allergic asthma via the action of different cytokines (IL-17, TGF-β, TNFα, and IL-1β) known to regulate lung damages and neutrophilic inflammation. These different cytokines can contribute to the pathogenesis of obesity-induced asthma exacerbation via various mechanisms. IL-1β, for example, has the capacity to contribute to lung inflammation via the recruitment of eosinophils, mast cells, dendritic cells and neutrophils [44]. TNFα, on the other hand, is known to be involved in obese asthmatics via its multiple deleterious actions on both immune cells (driving increased neutrophilic inflammation) and lung structural cells (driving abnormal lung mechanics such as airway hyper-responsiveness). The key role of TNFα in obesity is supported by several disease models, where conditions can be improved in obese mice by manipulating either TNFα or the TNF receptors [45]. Regarding TGF-β, increased levels have been reported in obesity and linked to enhanced airway inflammation, airway hyper-responsiveness and fibrosis. Indeed, previous studies showed that levels of TGF-β in both lung tissues and bronchoalveolar lavage fluids (BALFs) were increased in obese mice sensitised and challenged with the cockroach allergen [46]. Blocking TGF-β1 using neutralising antibodies reduced allergen-induced airway hyper-responsiveness and airway inflammation as well as lung tissue and perivascular fibrosis [47]. It is clear that enhanced cytokine production in obese asthma contribute to the aggravated disease in these patients.

The increased neutrophilic infiltration seen in lungs of obese mice is very interesting although the underlying mechanisms have not been clearly established. Clinical studies found the increased neutrophils in induced sputum of obese asthmatics suggesting a potential role of these cells in the severity of the disease [48,49,50,51,52]. Different cytokines were shown to play a key role in driving neutrophilic inflammation in asthma such as CXCL8, IL-17A or TNFα [53]. Interestingly, we found that the gene expression of *Tnfa*, *Il-1b*, *Tgf-b* and *Il17* cytokines were significantly increased in the lungs of obese asthmatic mice. Notably, the link between obesity and IL-17 was established in clinical studies [54], with Chen, Qin [55], for example, reporting a positive correlation between increased IL-17 levels and airway neutrophilia in stable obese individuals. Increased IL-17 expression in lung tissues was recently reported in experimental obese rats sensitised and challenged with OVA [56]. In addition, neutrophilic asthma has been related to increased TNFα levels [57,58], which was also reported in a obese mice OVA model [14]. These studies show that multiple mechanisms associated with obesity could be involved in promoting a neutrophilic inflammation profile in obese asthmatics. Although we showed an abnormal Th17–neutrophil axis in obese allergic asthma, the present study has some limitations like the small sample size. Because we did not own the flexivent system, we did not investigate the impact of obesity on allergen-induced airway hyper-responsiveness (AHR) in our model. Although there is strong evidence from previous studies showing that obesity exacerbates methacholine responses in both the OVA [14,39,40] and HDM [23,59] models, it is likely that similar lung function responses would be seen in our HDM model. Future studies could be performed to address this question.

Although we are convinced about the impact of HFD on allergic airway inflammation observed in our study using *n* = 4–9 mice/group, the conclusions were only based on a single study due to both financial and time constraints. Therefore, caution should be exercised before making a general statement and independent studies would be required to make a definitive statement about the overall impact of obesity on allergic asthma. Nonetheless, we feel confident about our conclusions since our approach was mostly based on the experimental approach used by several previous preclinical studies that have made interesting observations about the impact of obesity on asthma [15,16,17,18,19,20,22,39,40,60]. These studies, although diverse in their experimental protocols of diet-induced obesity and allergen sensitisation/challenge, used a quite similar range of animal number varying from *n* = 4 to 10 mice/group, with conclusions mostly derived from a single repeat as no mention was made regarding the internal validation of the studies. One elegant study did replicate their conclusions in three different experiments, and statistical analysis did confirm that the impact of obesity on the features of allergic asthma (i.e., airway hyper-responsiveness) could be seen in a number as low as *n* = 5 mice/group [14]. In fact, changes in allergen-induced TNFα levels in lungs between the HFD vs. ND groups seen in this study and by Andre, Calixto [60] were comparable. 

This preclinical study further supports a greater role of neutrophils in driving obesity-induced asthma severity possibly through their role in reducing patients’ response to corticosteroid therapy [49,61,62]. Indeed, several clinical studies found that a marked neutrophilic inflammation was associated with poor quality of life and poor response to current therapies including corticosteroids [63,64]. Thus, our study suggests the concept that drugs aimed at cytokines, such as IL-17 and TNFα, known here to correlate with neutrophilic inflammation in obese asthma, may lead to novel therapeutic interventions in these patients. 

## 4. Methods

### 4.1. Animals

The experimental use of mice in this study was approved by the UK Home Office (project license number: P43308E3B, Dr Stover is the project license holder). C57BL/6 male and female mice (purchased from Jackson laboratory) were obtained from a colony maintained at the University of Leicester’s Preclinical Research Facility. 

### 4.2. The Sensitisation and Challenge Protocol for Allergic Asthma in Obese (HFD) and Lean (ND) Mice

Mice that were 14–15 weeks old were randomised into two groups: a normal diet group (ND), mice fed with standard chow (Teklad 18% protein, Envigo), and a high-fat diet (HFD) group, who were fed chow with 42% of calories derived from fat (TD.88137, Envigo) for 10 weeks. On week 8 (day 1 of the sensitisation/challenge protocol), mice were first sensitised intranasally with house dust mite extracts (HDM) (25 µg of *D. pteronyssinus*, Citeq Biologics, Groningen, The Netherlands), followed by 5 additional nasal instillation doses of 25 µg HDM on days 4, 6, 11, 13, and 19 for the challenge steps. HDM extracts were prepared according to Citeq Biologics instructions, diluted in sterile PBS. The allergenic composition of HDM extract is mainly Der p1, endotoxin level 1.0 × 10^8^ u/g. Control mice received PBS only. The choice of HDM dose was based on previous reports describing the ability of 25 μg to reproduce the key features of allergic asthma [65,66,67,68]. The duration of HDM was based on our previous study (Supplementary Materials).

### 4.3. Lung Histology

Fixed lungs were paraffin embedded at Leicester Royal Infirmary, UK. The 4 μm lung sections were stained with Haematoxylin and Eosin (H&E) and scored in a double-blinded fashion using a light microscope, LaborLux S microscope (Leitz, Germany), to assess the grade of lung inflammation surrounding airways, blood vessels and lung parenchyma. Two lung sections per mouse with a total of 10 random fields at 10× magnification (5 random fields/section) were used to score the degree of inflammatory cell infiltration using a 3-scale grading system as follows: **0** (normal for absence), **1** (mild for some cell infiltrates in lung parenchyma), **2** (moderate for cell infiltrates around vessels and bronchi), and **3** (severe for extensive cell infiltrates around vessels and bronchi) [25,26,27]. Periodic acid-Schiff (PAS) stain was used to assess goblet cell hyperplasia and mucus production. The extent of airway mucopolysaccharide positivity was scored by two independent assessors with a score ranging from 0 (none) to 3 (full of mucin) as described [26,69,70,71] (Supplementary Materials). Hematologic Stain from Abcam, Cambridge, UK (ab150665) was used to stain eosinophils [72,73,74].

### 4.4. Immunohistochemistry Staining

Lung sections were de-paraffinised and hydrated, and heat-mediated antigen retrieval (citrate buffer) was performed for CD4^+^ and CD8^+^ staining. The lung sections were blocked with goat serum, and then incubated overnight with the primary antibody. Slides were washed three times by PBS, incubated with 3% H_2_O_2_ for 15 min to block endogenous peroxidase, then washed with PBS, before a secondary antibody was added for 20 min followed by three times washing steps. Finally, DAB (3,3′-diaminobenzidine) reagent was added for 5 min, then washed under running tap water before being dehydrated with industrial methylated spirit (IMS) followed by xylene and finally being mounted with DPX mounting solution (Sigma-Aldrich: St. Louis, MO, USA). The primary antibody to stain neutrophils was rat monoclonal anti-neutrophil antibody (Cat number: ab2557, Abcam) and detected with secondary goat anti-rat IgG-HRP (Cat number: sc-2006, Santa Cruz Biotechnology, Dallas, TX, USA). The primary antibodies against T lymphocyte markers were rabbit monoclonal to CD4 (Cat number: ab183685, Abcam) and rabbit monoclonal to CD8 (cat number: ab183685, Abcam), both detected with secondary goat Anti-rabbit IgG polymer kit (MP-7451) from Vector Laboratories.

### 4.5. Quantification of Total IgE and HDM-Specific IgE in Serum

Total IgE was measured using ELISA as mentioned [24]. Briefly, a 96-well plate was coated with 1 µg/mL goat anti-mouse IgE (cat number: ab19967, Abcam) in PBS overnight at 4 °C. The next day, the plate was washed three times with washing buffer and blocked for 1 h with 1% BSA, and washed again, before diluted (1:10) serum samples were added overnight at 4 °C. The next day, the plate was washed three times, before a biotinylated rat anti-mouse IgE (clone R35-72, Cat 553414, BD Pharmingen) from BD Biosciences, San Diego, CA, USA was added and incubated for 2 h at room temperature. Streptavidin-Peroxidase was added to each well, incubated for 30 min at room temperature before the substrate (Sigma-Aldrich) was added and the plate was read at 450 nm. 

HDM-IgE level was determined as recommended by the Citeq Biologics Biotinylated-HDM Der P1 IgE ELISA protocol. A 96-well plate was coated with 100 µL per well anti-mouse IgE (Citeq part number, HE.02.01) capture antibody diluted in coating buffer. Then, the plate was incubated overnight at 4 °C. The next day, the plate was washed three times with washing buffer and blocked for 1 h, then washed with PBS. Serum samples were incubated at room temperature for 2.5 h. After the plate was washed three times, samples were labelled with biotinylated HDM/Der p 1 (Citeq part number, 02.01.88) and incubated for 1 h at room temperature. Streptavidin-Peroxidase was added to each well, and then incubated for 30 min at room temperature. The plate was read at 450 nm.

### 4.6. Quantitative PCR

qPCR analysis was performed as described previously [70]. Primers were ***Il5***, forward 5′-TCACCGAGCTCTGTTGACAA-3′ and reverse 5′-CCACACTTCTCTTTTTGGCG-3′; ***Il13***, forward 5′-TGAGGAGCTGAGCAACATCACACA-3′ and reverse 5′-TGCGGTTACAGAGGCCATGCAATA-3′; ***Il17***, forward 5′-GGCTGACCCCTAAGAAACC-3′ and reverse 5′-CTGAAAATCAATAGCAGGAAC-3′; ***Tgf-b***, forward 5′-CACGTAGTAGACGATGGGCA-3′ and reverse 5′-TTTAGGAAGGACCTGGGTTG-3′; ***Il-1b***, forward 5′-TCGCTCAGGGTCACAAGAAA-3′ and reverse 5′-CATCAGAGGCAAGGAGGAAAA-3′; ***Tnfa***, forward 5′-ACATTGGAGGCTCCAGTGAATTCGG-3′ and reverse 5′-GGCAGGTCTACTTTGGAGTCATTGC-3′.

### 4.7. Statistical Analysis

Data are presented as the mean ± SEM. Comparison between 2 groups was made using an unpaired *t*-test (two-tailed) while multiple group comparisons were made using one-way ANOVA, followed with Sidak’s multiple comparisons test. Statistical analyses were conducted using GraphPad Prism (version 9.2.0; GraphPad Software).

## Data Availability

The data presented in this study will be made available upon request from the corresponding author.

## Supplementary Materials

The following supporting information can be downloaded at: https://www.mdpi.com/article/10.3390/ijms25116170/s1.
