# Supplementary material for: Obesity Enhances Non-Th2 Airway Inflammation in a Murine Model of Allergic Asthma
Source: Int J Mol Sci. 2024 Jun 4;25(11):6170. doi: 10.3390/ijms25116170 (PMC11172812; doi:10.3390/ijms25116170)
Supplement: Supplementary file 1 [file ijms-25-06170-s001.zip › ijms-2996955-supplementary.pdf]

Supplemental data

Table S1. inflammation score system used in this study.

Score 0	Score 1	Score 2	Score 3
**No inflammation** no inflammatory cell infiltration, normal lung tissue observed.	**Mild inflammation** some inflammatory cell infiltrates in lung parenchyma (≤ 3 cells deep).	**Moderate inflammation** inflammatory cell infiltrates around vessels and bronchi (> 3 cells deep).	**Severe inflammation** – Dense inflammatory cell infiltration around blood vessels and bronchi. Cellular infiltration is widespread in lung parenchyma with little area for gas exchange (>6 cell deep).

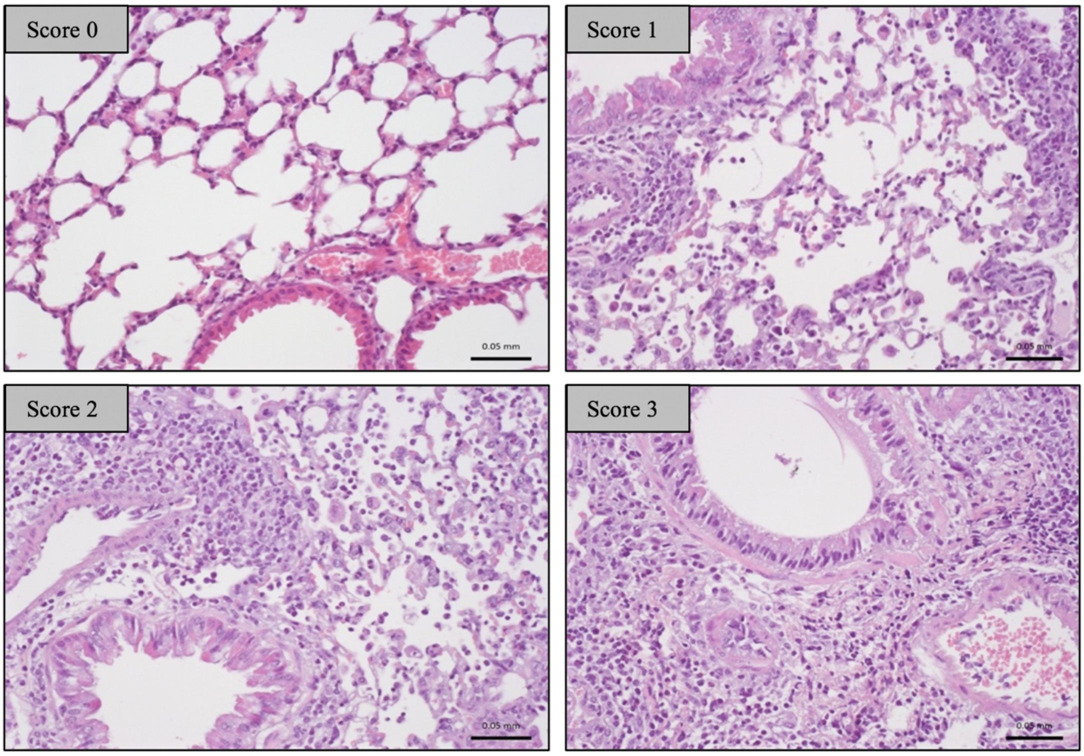

Figure S1. Representative lung sections of the inflammation scoring system. A 3-scale grading system was used to score for the degree of inflammatory cell infiltration as followed: **0** (*normal* for absence), **1** (*mild* for some cell infiltrates in lung parenchyma), **2** (*moderate* for cell infiltrates around vessels and bronchi), **3** (*severe* for extensive cell infiltrates around vessels and bronchi.

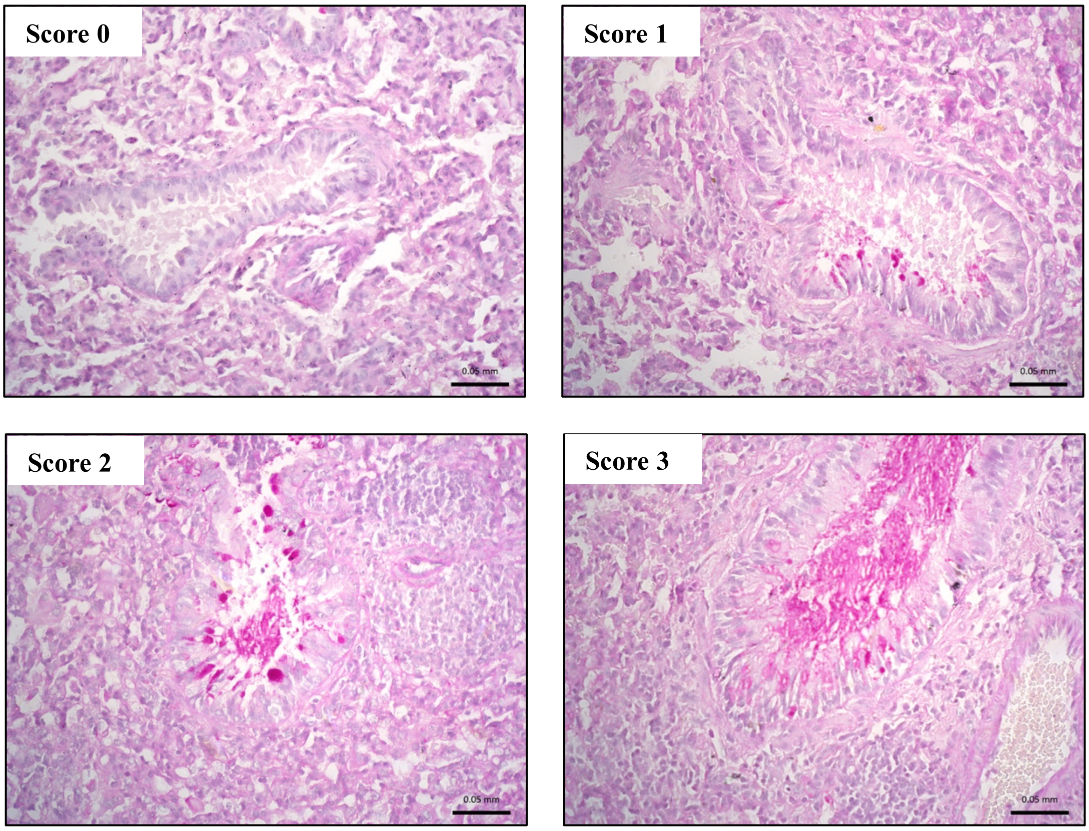

Figure S2. Representative lung sections of PAS scores. Numerical scores from 0 to 3 for the abundance of PAS-positive goblet cells in each airway were determined as follows: **Score** 5% goblet cells), **1** (5 to 25% goblet cell), **2** (25 to 75% goblet cells), **3** (> 75% goblet cells).

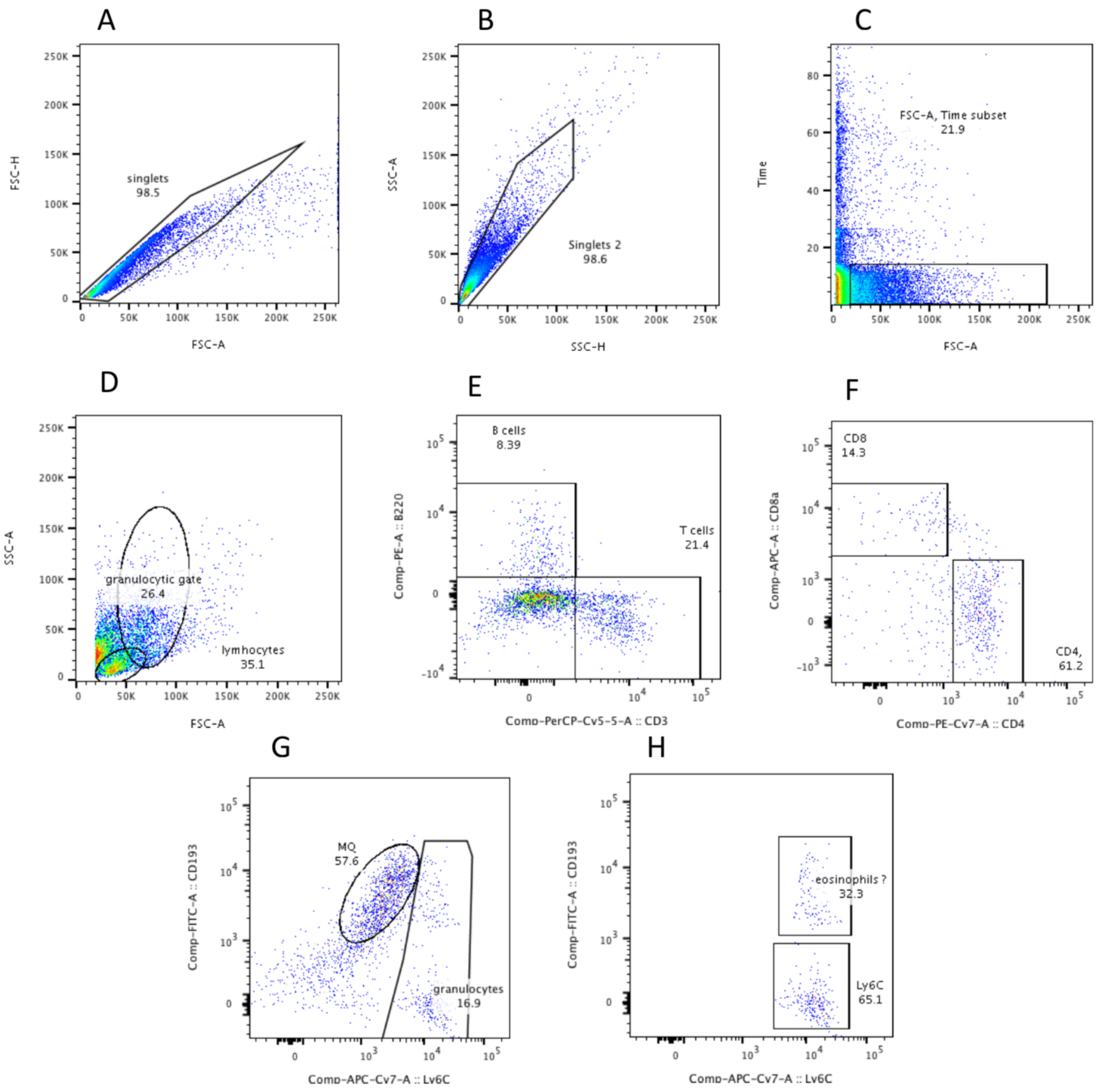

Figure S3. Determining immune cell population in single cell lung suspension. **A-C**, Live cells were gated by removing cellular debris and doublets with low forward (FSC) and side scatters (SSC). Then data cleaned by time filter, **C**.**D-F**, Lymphocytes were gated using known forward scatter (FSC-A) and side scatter (SSC-A) in **D**, which is proportional to cell size and granularity, respectively. **E**, from lymphocyte gate, B cells were identified using anti-mouse CD45R/B 220 -PE and T cells using anti-mouse CD3ε PE/Cyanine5 and gated accordingly. **F,** T cell subsets: CD4 and CD8 were determined from T cell gate ($CD3^+$ cells) and identified by CD4 PE- Cyanine 7and CD8 APC. **G and H**, Granulocyte were gated using known forward scatter (FSC-A) and side scatter (SSC-A) in **D** granulocytic gate. Then eosinophils and neutrophils were identified using FITC and APC-CY7.

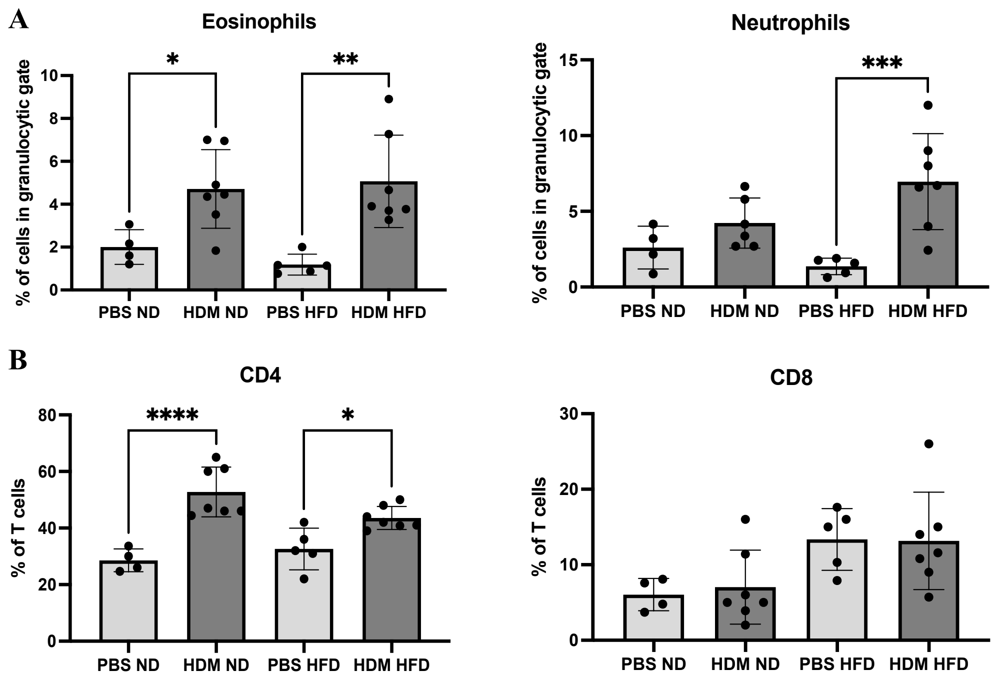

Figure S4. Assessment of immune cell population in single cell lung suspension using Flow cytometry. A, percentage of granulocytes, eosinophils were increased after HDM sensitisation and challenge (** *p*<0.005 * *p*<0.05), neutrophils were significantly increased in high fat diet fed mice exposed to HDM (HDM HFD, ****P* < 0. 001). **B**, percentage of T cells, CD4$^+$ T cells were significantly increased after HDM sensitisation and challenge (**** *p*<0.0001 * *p*<0.05), HFD groups had higher percentage of CD8$^+$ T cells. Data represent the mean ± SEM (n=4-7). ANOVA was used to compare between groups with each circle representing a different mouse.

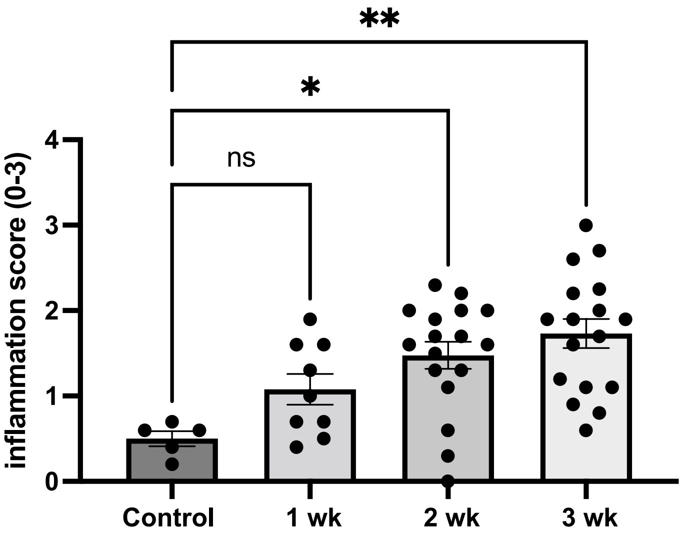

Figure S5. **Time course of HDM exposure on lung inflammation.** C57BL/6J mice were sensitised and challenged with intranasal house dust mite (HDM) while being fed with either HFD (42% of Kcal fat) or normal mice chow (18% Kcal fat) for 7 weeks. Lung inflammation showed a significant increase with HDM duration in the 2- and 3-week models irrespective to diet. Data represent the mean ± SEM in n=5-17 animals. ANOVA was used to compare between groups with each circle representing a different mouse.
